# 7-*epi*-Clusianone, a Multi-Targeting Natural Product with Potential Chemotherapeutic, Immune-Modulating, and Anti-Angiogenic Properties

**DOI:** 10.3390/molecules24234415

**Published:** 2019-12-03

**Authors:** Wesley F. Taylor, Maria Yanez, Sara E. Moghadam, Mahdi Moridi Farimani, Sara Soroury, Samad N. Ebrahimi, Marzieh Tabefam, Ehsan Jabbarzadeh

**Affiliations:** 1 Department of Chemical Engineering, University of South Carolina, Columbia, SC 29208, USA; wftaylor@email.sc.edu (W.F.T.); yanez@mailbox.sc.edu (M.Y.); eslambol@mailbox.sc.edu (S.E.M.); 2 Department of Phytochemistry, Medicinal Plants and Drugs Research Institute, Shahid Beheshti University, G. C., Evin, Tehran 19839-69411, Iran; m_moridi@sbu.ac.ir (M.M.F.); s_ebrahimi@sbu.ac.ir (S.N.E.); m_tabefam@sbu.ac.ir (M.T.); 3 Department of Phytochemistry, Faculty of Science, Golestan University, Gorgan 15759-49138, Iran; sarasoroury@gmail.com; 4 Biomedical Engineering Program, University of South Carolina, Columbia, SC 29208, USA

**Keywords:** cancer, natural compounds, immunomodulation, chemotherapeutics, multitargeting

## Abstract

Targeted therapies have changed the treatment of cancer, giving new hope to many patients in recent years. The shortcomings of targeted therapies including acquired resistance, limited susceptible patients, high cost, and high toxicities, have led to the necessity of combining these therapies with other targeted or chemotherapeutic treatments. Natural products are uniquely capable of synergizing with targeted and non-targeted anticancer regimens due to their ability to affect multiple cellular pathways simultaneously. Compounds which provide an additive effect to the often combined immune therapies and cytotoxic chemotherapies, are exceedingly rare. These compounds would however provide a strengthening bridge between the two treatment modalities, increasing their effectiveness and improving patient prognoses. In this study, 7-*epi*-clusianone was investigated for its anticancer properties. While previous studies have suggested clusianone and its conformational isomers, including 7-*epi*-clusianone, are chemotherapeutic, few cancer types have been demonstrated to exhibit sensitivity to these compounds and little is known about the mechanism. In this study, 7-*epi*-clusianone was shown to inhibit the growth of 60 cancer cell types and induce significant cell death in 25 cancer cell lines, while simultaneously modulating the immune system, inhibiting angiogenesis, and inhibiting cancer cell invasion, making it a promising lead compound for cancer drug discovery.

## 1. Introduction

In recent years, new hope has been given to patients diagnosed with cancer due to the emergence of targeted therapeutics [1]. However, due to the limitations of newly discovered targeted therapies, cancer remains the second leading cause of death in the United States according to the Centers for Disease Control and Prevention [2,3]. Of the approximately 610,000 deaths caused by cancer in 2018, over 25% will be due to lung and bronchus cancers, making them the deadliest cancer type in the United States [4]. The use of targeted therapies has represented a paradigm shift from traditional chemotherapeutics (often derived from multi-targeting natural products) to molecules and antibodies affecting specific cellular functions. Within the category of targeted therapies, immune modulating therapies have been the source of many novel treatments. Monoclonal antibodies [5], cytokines [6], dendritic cell therapies [7], chimeric antigen receptor T cells (CAR-T cells) [8], and immune checkpoint blockade therapies [9] are among the immune modulating treatments that have been approved by the Food and Drug Administration (FDA) for the treatment of cancer.

Each of these emerging technologies has led to an increase in treatment responses and survival times, however, major drawbacks have been identified for each. For example, the dendritic cell therapy, sipuleucel-T, has been shown to be effective in treating early metastatic castration-resistant prostate cancer, but is extremely costly due to the personalized nature of the treatment [10]. CAR-T cell therapies similarly function by genetically engineering T cells from a patient to target cancer cells, but come with a record setting $475,000 price tag, neurotoxicities, and cytokine release syndrome [11,12]. Other immune regulating treatments, including immune checkpoint blockade therapies such as nivolumab, tend to be more cost effective but less personalized to each patient [13]. As a result, these treatments are often used in combination with other therapies. Modulation of the activity of macrophages found within the tumor microenvironment has also garnered interest as a potential immune regulating cancer therapy, though the FDA has not currently approved a macrophage-targeted therapy [14].

Targeted therapies used to disrupt the unregulated growth signals of mutated proto-oncogenes or disrupt angiogenesis have similarly changed the landscape of cancer treatment despite being accompanied by major drawbacks. Angiogenesis targeting therapies such as bevacizumab and sorafenib have been used to treat solid and metastatic tumor growth, but similarly suffer from acquired resistance, likely as a result of plasticity of the tumor microenvironment [15]. The disappointing performance of targeted therapies has led to a renewed interest in multi-targeting natural products [16].

Natural products have a long history of use as cancer therapies [17], either being used for or inspiring approximately 60% of cancer treatments used between 1981 and 2006 [18]. These compounds tend to be safe and of low cost in addition to targeting a number of cellular functions simultaneously. By targeting multiple cancer related pathways, natural compounds may be able to provide a robust, widely applicable treatment less susceptible to resistance [19]. Natural products targeting the immune response in addition to inducing cancer cell death are uniquely positioned to synergize well with current treatment regimens [20]. Compounds of this type may be able to limit the number of immune therapies and chemotherapies used in cancer treatment cocktails in addition to increasing their efficacy [21]. The number of compounds with combined chemotherapeutic and immune-modulatory effects is limited, and many of these compounds are classified as natural products [22]. Notable examples include astragaloside IV [23,24], curcumin [25], emodin [26,27], and total saponins of panax ginseng [28,29]. These compounds have been demonstrated to induce apoptosis in cancer cells while simultaneously altering cytokine expression or macrophage polarization. However, the low bioavailability and limited efficacy of these compounds warrants further drug discovery efforts for multi-targeting compounds.

7-*epi*-clusianone and its configurational isomers are natural products that have been demonstrated to feature antimicrobial, anti-allergenic, schistosomicidal, and anti-inflammatory effects [30,31,32,33]. Additionally, these compounds have been shown to induce cell death in glioblastoma, as well as lung, melanoma, breast, prostate, renal, cervical, and tongue cancer [34,35,36,37]. Researchers have identified several potential molecular targets of 7-*epi*-clusianone, including microtubules, the mitochondrial membrane, cathepsins, and cyclins [34,36,37,38]. It is likely that multiple molecules are targeted by 7-*epi*-clusianone and its isomers due to the so-called “privileged structures” typically possessed by natural products. Limited in vivo studies have demonstrated the safety of a 7-epiclusianone in vivo in administrations of up to 300 mg/kg of body weight in female albino mice [30]. Additionally, the chemical synthesis of 7-*epi*-clusianone has been explored, allowing for a high availability of the compounds in the future [39]. As a result, 7-*epi*-clusianone is a promising lead compound for multi-targeted cancer treatment. However, screening of 7-*epi*-clusianone across a wide range of cancers has not yet been reported. Additionally, only limited studies of the molecular targets of 7-*epi*-clusianone have been performed, and the direct interaction of 7-*epi*-clusianone and its purported targets has yet to be determined. Finally, the effect of 7-*epi*-clusianone on the anticancer immune response has not been explored. To address these knowledge gaps, herein the combined anticancer and immune modulatory effects of 7-*epi*-clusianone are examined.

## 2. Results

### 2.1. 7-epi-clusianone Inhibits Cell Growth and Induces Cell Death in a Wide Spectrum of Cancers

**Figure 1:**
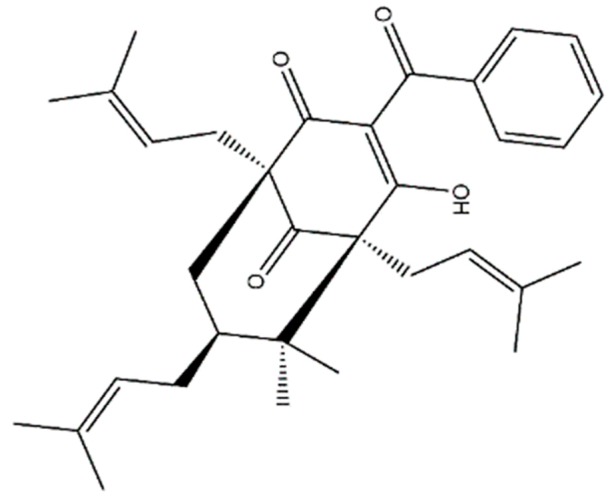
The structure of the polycyclic polyprenylated acylphloroglucinol, 7-*epi*-clusianone.

To determine the cytotoxic potential of 7-*epi*-clusianone (Figure 1), the compound was screened against the 60 cancer cell lines of the National Cancer Institute’s NCI-60 cancer panel.

For each of the cell lines in the panel, the concentration of compound required to reduce the growth of cells to 50% that of the vehicle control (GI50), the concentration required to inhibit the growth of any amount of cells (TGI), and the concentration required to induce cell death of 50% of the seeded cells (LD50) was determined (Figure S3).

7-*epi*-clusianone inhibited the growth of all 60 cell lines in the NCI-60 cancer panel (Figure S3-A) with an average GI50 of 2.7 µM. 7-*epi*-clusianone did not, however, exhibit a high level of selectivity in inhibiting the growth of the cancer cell lines as the determined GI50 concentrations only ranged from 1.63 µM to 3.89 µM. The selectivity of 7-*epi*-clusianone was higher in completely inhibiting cell growth. The majority of TGI for cell lines within the panel were less than 10 µM, however, two leukemia cell lines continued concentrations to grow after 48 h of incubation with 101 µM of 7-*epi*-clusianone, the highest concentration tested (Figure S3-B). In addition to inhibiting the growth of many cancer cell lines, 7-*epi*-clusianone exhibited an LD50 of less than 101 µM for 25 of the cell lines tested (Figure S3-C). Interestingly, the cytotoxicity of 7-*epi*-clusianone varied widely within each tissue type, with the exception of leukemia, for which no LD50 under 101 µM was observed.

**Figure 2:**
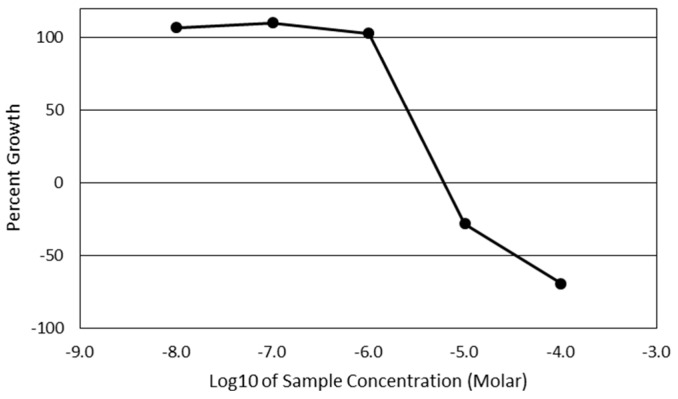
Percent growth of NCIH460 non-small cell lung cancer cells after 48 h of treatment with 7-*epi*-clusianone in the NCI-60 panel. The NCI-60 panel is a sulforhodamine B based screening method of 60 immortalized cancer cell lines. The GI50, TGI, and LD50 concentrations of 7-*epi*-clusianone for the NCIH460 cells were determined to be 2.6 µM, 6.2 µM, and 35 µM, respectively.

The full growth response data for each of the cell lines can be found in Table S1. As lung and bronchus cancers are projected to be responsible for over one quarter of all cancer-related deaths in the United States in 2018, and therefore possess a higher potential for clinical impact compared to other tissue types in the NCIH460 panel, the effect of 7-*epi*-clusianone on small cell lung cancer was investigated further. The non-small cell lung cancer cell line, NCIH460, was the most sensitive lung cancer cell line to 7-*epi*-clusianone when considering induced cell death (LD50) and was chosen for further investigation as a result. The GI50, TGI, and LD50 concentrations of the NCIH460 cell line were 2.6 µM, 6.2 µM, and 35 µM, respectively (Figure 2).

### 2.2. 7-epi-clusianone Induces G1 Arrest Followed by Apoptosis in NCIH460 Small Cell Lung Cancer Cells

**Figure 3:**
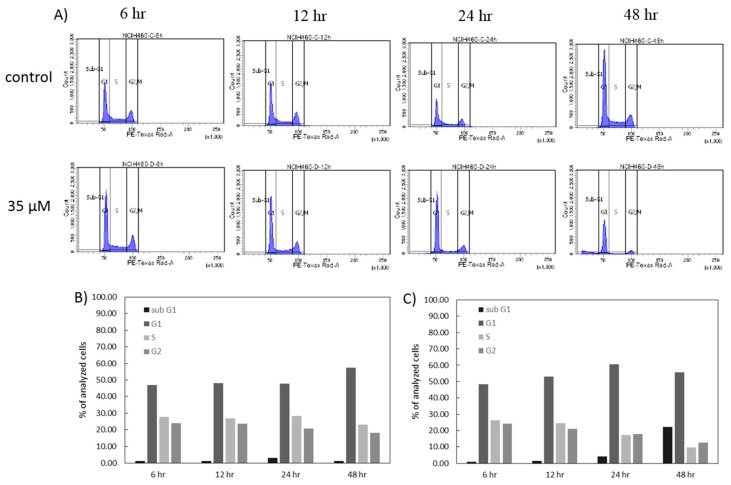
Cell cycle flow cytometry experiment showing the (**A**) histogram of propidium iodide expression of NCIH460 cells after four different time points of treatment with either a vehicle control or 7-*epi*-clusianone. The histograms were divided into 4 regions representing the sub-G1, G1/G0, S, and G2/M phases of the cell cycle. The percentage of cells in the sub-G1, G1/G0, S, and G2/M regions is shown for both (**B**) the cells treated with the vehicle control and (**C**) the cells treated with 35 µM of 7-*epi*-clusianone.

In order to gain insight into the mechanism of action of 7-*epi*-clusianone on NCIH460 small cell lung cancer cells, the cell cycle of the NCIH460 cells over 48 h under exposure to 7-*epi*-clusianone was analyzed by quantifying the amount of PI bound to DNA within the cells using flow cytometry (Figure 3A–C). When the NCIH460 cells were treated with 35 µM of the compound the number of cells in the G1 phase of the cell cycle increased at 12 and 24 h of incubation when compared to the vehicle control. Additionally, a depletion of cells in the S and G2/M phases of the cell cycle occurred after 24 h when compared to the control and persisted after 48 h. The percent of cells in the S phase decreased at a rate faster than the percent of cells in the G2/M phase decreased when exposed to 7-*epi*-clusianone, suggesting that cells were progressing from the S phase to G2/M phase, but they were unable to progress from the G1 phase to S phase when exposed to 7-*epi*-clusianone. At the 48-h time point, the percentage of cells in the G1 phase decreased when compared to the percent of cells in the G1 phase at 24 h when exposed to 7-*epi*-clusianone. As the percent of cells in the S and G2/M phases continued to decrease over this same time period, this decrease in G1 phase cells can be explained by the increase in sub G1 cells seen from 24 h to 48 h of incubation with 7-*epi*-clusianone. These sub G1 cells are hypothesized to be dead cells with cleaved DNA.

**Figure 4:**
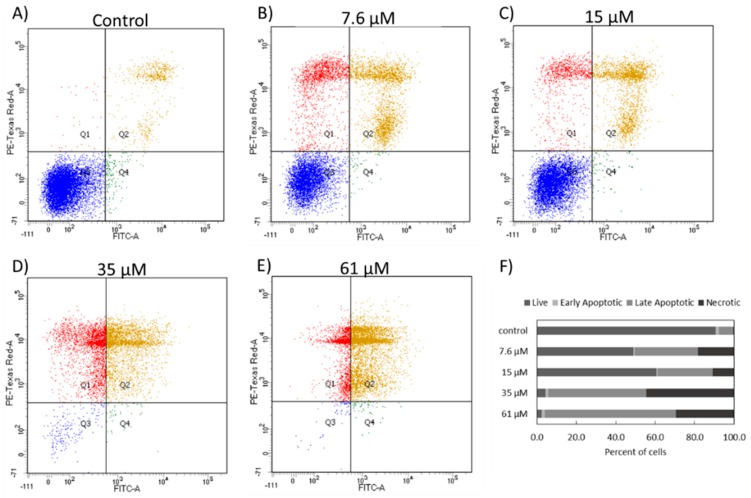
Annexin V/propidium iodide expression of NCIH460 cells exposed to (**A**) a vehicle control, or different concentrations of 7-*epi*-clusianone, (**B**) 7.6 µM, (**C**) 15 µM, (**D**) 35 µM, and (**E**) 61 µM for 48 h. The expression scatterplots were divided into four quadrants representing double negative cells, annexin V positive cells, propidium iodide positive cells, and double positive cells. Double negative cells were considered live cells, annexin V positive cells were considered early apoptotic cells, propidium iodide cells were considered necrotic, and double positive cells were considered late apoptotic cells. The divided histograms were used to express the percentage of cells that were live, early apoptotic, late apoptotic, or necrotic within each treatment group (**F**).

Annexin V/PI expression for the NCIH460 cells after 48 h of incubation with 7.6 µM, 15 µM, 35 µM, and 61 µM 7-*epi*-clusianone was determined to evaluate whether the cells observed in the sub-G1 phase had undergone apoptosis (Figure 4). As the concentration was increased the number of live cells decreased, confirming the dose dependent cytotoxicity of 7-*epi*-clusianone on NCIH460 cells. In each of the 7-*epi*-clusianone-treated groups, the number of apoptotic and necrotic cells increased when compared to the vehicle control (Figure 4A). The number of apoptotic, Annexin V positive, cells increased as the concentration of 7-*epi*-clusianone was increased. Additionally, at lower concentrations, a population of PI positive and Annexin V low cells was observed (Figure 4B,C). This suggests that at lower concentrations, a population of NCIH460 cells were just beginning to permeabilize and therefore were in an earlier stage of apoptosis after 48 h than were cells treated with a higher dose of 7-*epi*-clusianone (Figure 4D–F).

**Figure 5:**
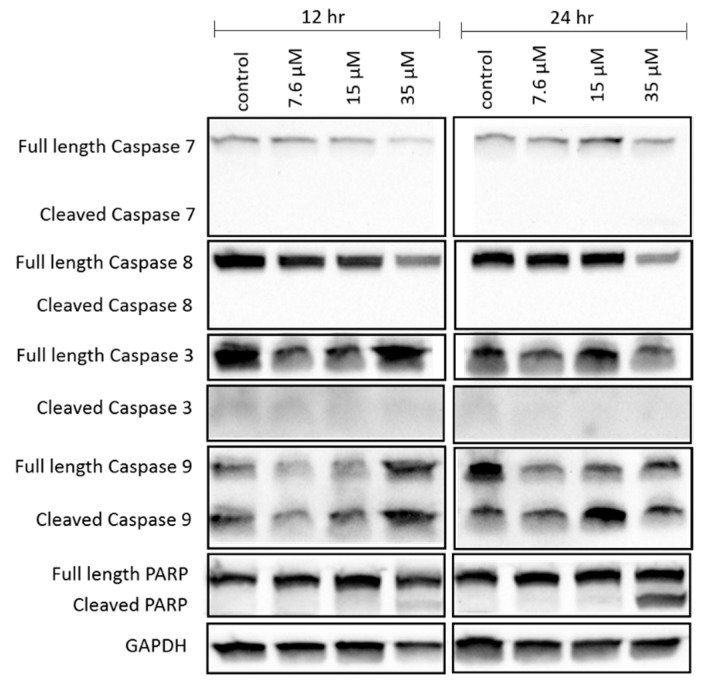
Expression of apoptosis-related proteins in NCIH460 cells after treatment with 7-*epi*-clusianone as determined by electrophoresis followed by western blot analysis. Cells were treated with 7-*epi*-clusianone for 12 or 24 h before loading 50 µg of isolated protein into each well. Images were taken from the same sample run in duplicates and 2 different gels. Gel 1 was stained for Caspase 3 and 9. Gel 2 was stained for Caspase 7 and 8, PARP, and GAPDH. The contrast and brightness of the images were adjusted using Image Lab software from Bio-Rad. The cropped gels are displayed in this figure and the full-length of blots are presented in Supplementary information Figure S5.

We also determined the caspase activity of the NCIH460 cells to support the hypothesis that apoptosis occurs after exposure to 7-*epi*-clusianone. The quantity of inactive/full length and active/cleaved forms of caspases 7, 8, 3, and 9 present with the NCIH460 cells was determined using western blotting (Figure 5). After 12 and 24 h of exposure to 7-*epi*-clusianone, the full length form of caspases 7 and 8 decreased as the concentration was increased, but the cleaved forms of these caspases were not detected. No trend was apparent from the quantity of caspase 3 detected at either time point. However, the ratio of cleaved caspase 9 to full length caspase 9 increased dramatically for NCIH460 cells treated with 7.6 µM or 15 µM of 7-*epi*-clusianone for 12 or 24 h. Additionally, a dose- and time-dependent increase in the ratio of cleaved Poly (ADP-ribose) polymerase (PARP) to full length PARP occurred under incubation with 7-*epi*-clusianone. The cleavage of caspase 9 and PARP further supports that 7-*epi*-clusianone induces apoptosis in NCIH460 lung cancer.

### 2.3. 7-epi-clusianone Inhibits Angiogenesis and Cell Migration of NCIH460 Cells

**Figure 6:**
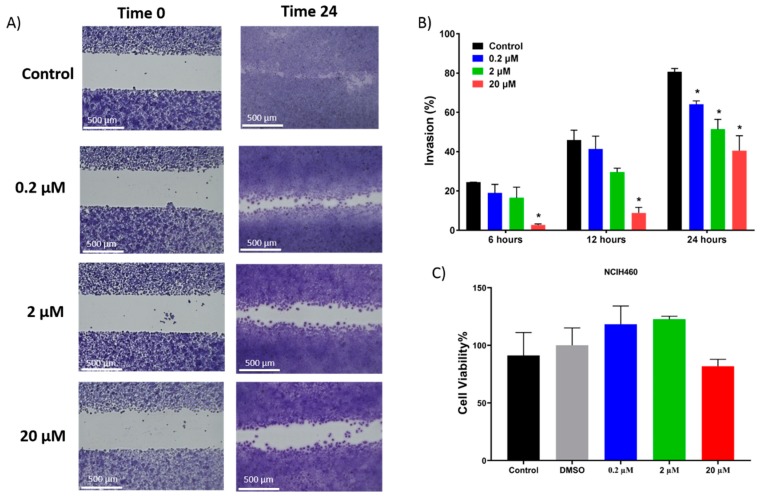
Invasion of NCIH460 cells into a cell free gap. Cells were treated with either a vehicle control or 7-*epi*-clusianone for 24 h. (**A**) Representative images of each treatment group after 24 h in addition to (**B**) graphs depicting the % invasion of each treatment group after 6, 12, and 24 h. (**C**) The viability of NCIH460 cells after 24 h of incubation with 7-*epi*-clusianone was determined using MTS assay. All data are statistically presented as the mean ± standard error. Multiple t-tests were performed using Graph-Pad Prism 7.03 (GraphPad, La Jolla, CA, USA) to determine the significance between each experimental group. *p* values of less than 0.05 were considered to be significant (* denotes significant difference compared to the control group in the same time point).

After determining 7-*epi*-clusianone’s ability to induce cell death at high concentrations, the ability of 7-*epi*-clusianone to inhibit cell migration of NCIH460 cells at low concentrations was assessed. A uniform cell gap was formed using cell culture insert, and the cells were allowed to grow for up to 24 h exposed to either a vehicle control or 7-*epi*-clusianone (Figure 6A,B). After 24 h, the cell-free gap had almost completely closed for cells treated with the vehicle control. In contrast, after 24 h of incubation with 7-*epi*-clusianone the percent invasion of NCIH460 cells was significantly lower compared to the control for each concentration used, including 200 nM, a concentration 10-times lower than the GI50 concentration. At each time point considered (6, 12, and 24 h), a dose dependent decrease in invasion was observed. Also, the toxicity effects of 7-*epi*-clusianone was evaluated by tracking the NCIH460 viability at 24 h using MTS assay. No significant difference (*p* ≤ 0.05) in cell viability was observed when we compared the treated cells with blank control group (Figure 6C). This indicates that the reduction in cell invasion in the presence of higher compound concentrations is not affected by cell viability.

**Figure 7:**
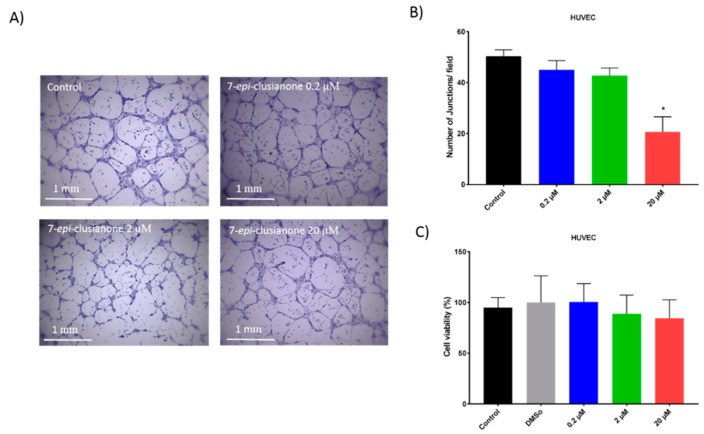
Tube formation between human umbilical vascular endothelial cells (HUVEC) endothelial cells after 8 h of incubation on growth factor reduced BD Matrigel^TM^ with or without 7-*epi*-clusianone. A representative image of the cells treated with the vehicle control, 0.2 µM, 2 µM, and 20 µM 7-*epi*-clusianone is shown in part (**A**). The average number of junctions counted per field is graphed in part (**B**). The viability of HUVEC cells after 8 h of incubation with 7-*epi*-clusianone as determined by MTS assay is shown in part (**C**). *p* values of less than 0.05 were considered to be significant (* denotes significant difference compared to the control group in the same time point).

Similarly, the ability of 7-*epi*-clusianone to inhibit angiogenesis was indirectly assessed. Human umbilical vascular endothelial cells (HUVEC) endothelial cells were incubated with or without 7-*epi*-clusianone on a growth factor reduced BD Matrigel^TM^ basement matrix. Tube formation between the endothelial cells was assessed after 8 h (Figure 7A). A dose dependent reduction of tube formation was observed when the cells were exposed to 7-*epi*-clusianone at concentrations between 0.2 and 20 µM (Figure 7B). The reduction of tube formation became significant (*p* ≤ 0.05) at 20 µM 7-*epi*-clusianone. An MTS viability assay of HUVEC endothelial cells in response to 7-*epi*-clusianone for 8 h revealed that the compound was not toxic at this time point (Figure 7C). No significant difference in viability was observed in these cells even at the highest concentration of 20 µM 7-*epi*-clusianone.

### 2.4. 7-epi-clusianone Directly Targets Tubulin Polymerization, JAK3, and ALK (C1156Y)

**Figure 8:**
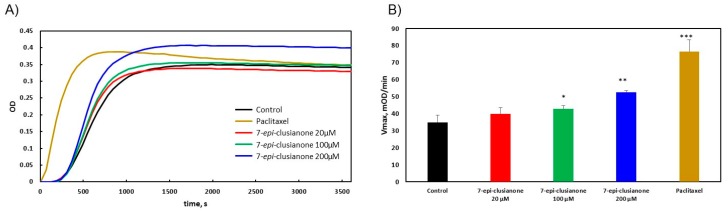
The extent of tubulin polymerization as determined by absorbance at 340 nm for 1 h. (**A**) The average absorbance of three repetitions of the tubulin polymerization assay treated with 7-*epi*-clusianone, paclitaxel, or a vehicle control, and (**B**) the average calculated Vmax for each treatment group (* represents *p* ≤ 0.05 compared to the control, ** represents 0.05 ≤ *p* ≤ 0.01 compared to the control, and *** represents 0.01 ≤ *p* ≤ 0.001).

While the cytotoxic effect of 7-*epi*-clusianone on a number of cell lines has been previously reported, the direct effect of 7-*epi*-clusianone on its purported molecular targets has yet to be determined [34,35,36,37]. Due to the identification of microtubules as a potential target of clusianone in other lung cancer cell lines [37], the effect of 7-*epi*-clusianone on tubulin polymerization was investigated using the cytoskeleton tubulin polymerization assay (Figure 8). While the nucleation time, i.e., the time required for tubulin to begin polymerizing, was not affected by the presence of 7-*epi*-clusianone, the rate at which the tubulin polymerized was significantly increased by 100 and 200 µM of 7-*epi*-clusianone (Figure 8A). The rate at which the tubulin polymerized is best represented by the Vmax value, which corresponds to the highest ratio of increase in the absorbance at 340 nm to time for each treatment group. We observed a dose dependent increase in Vmax, consistent with the increase in Vmax observed in the presence of the known microtubule stabilizing agent, paclitaxel [40] (Figure 8B).

To determine the ability of 7-*epi*-clusianone to target tyrosine receptor kinases, a common target of cancer therapeutics, the compound was screened using the DiscoverX scanTK kinase panel. This panel determines the ability of a compound to inhibit the activity of 135 different tyrosine kinases. A reduction of kinase activity to 30% or below is considered significant in this one dose screen. At a concentration of 20 µM, 7-*epi*-clusianone significantly inhibited two kinases, anaplastic lymphoma kinase C1156Y (ALK C1156Y) and janus kinase 3 (JAK3). The activity of ALK (C1156Y) and JAK3 were inhibited by 70% and 75% respectively. ALK (C1156Y) is a mutation of the ALK tyrosine kinase, which has been associated with acquired resistance to the targeted ALK inhibitor, crizotinab, used for the treatment of small cell lung cancer [41]. JAK3 is a kinase that plays a role in the immune response of several types of cells [42]. Kinase assay report is found in the supplementary information Table S2.

### 2.5. 7-epi-clusianone Increases the Expression of Pro-Inflammatory Cytokines in THP-1 Macrophages

**Figure 9:**
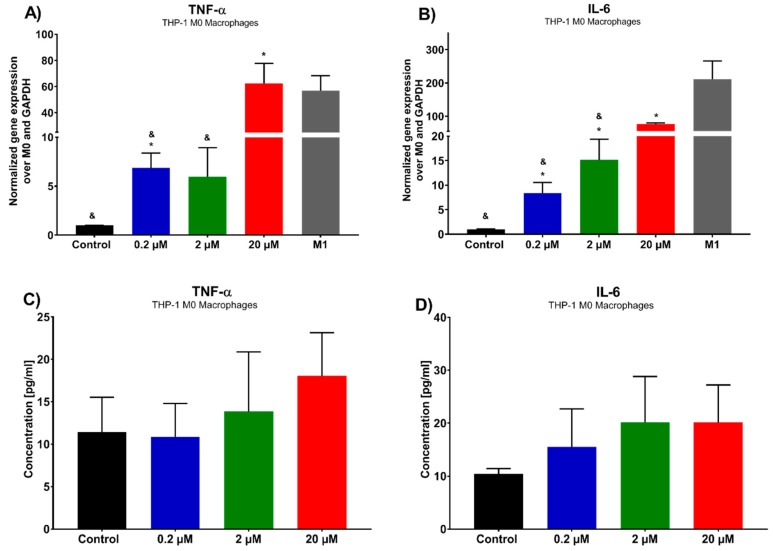
Cytokine expression of THP M0 macrophages after 72 h treatment with 7-*epi*-clusianone (**A**) TNFα and (**B**) IL-6 by rt-PCR. Gene expression was normalized to the housekeeping gene GAPDH and the control group, non-treated THP-1 M0 macrophages (2^−ΔΔC^). M1 macrophages were stimulated with LPS and IFN for 24 h and were characterized by rt-PCR to verify phenotypic markers (TNFα and IL-6). Cytokine concentration in culture media was determined for (**C**) TNFα and (**D**) IL-6 by ELISA assay. *p* values of less than 0.05 were significant. (* denotes significant difference compared to the control group and & denotates significant difference compared to the positive control (M1 macrophages)).

In order to determine the effect of 7-*epi*-clusianone on the immune response, cytokine concentration was assessed on M0 macrophages. A shift in M0, naïve macrophages, to M1 polarized, pro-inflammatory and anticancer macrophages, is denoted by an increase in inflammatory cytokines, including TNFα and IL-6 among others. We assessed the expression of these cytokines by THP-1 macrophages after 72 h of incubation with 7-*epi*-clusianone (Figure 9). In addition, using rt-PCR, we examined the phenotypic markers of M1 macrophages (TNFα and IL-6) as a positive control. Both the gene expression of the cytokines and the concentration of the cytokine excreted into the cell culture media were determined by rt-PCR and ELISA, respectively. To ensure that the concentrations of 7-*epi*-clusianone tested on macrophages were adequate, the viability and invasion capability of the macrophages under exposure to 7-*epi*-clusianone was tested and no significant changes were observed (Figure S4). The gene expression of both TNFα and IL-6 increased as the concentration of 7-*epi*-clusianone was increased (Figure 9A,B, respectively). At 20 µM of 7-*epi*-clusianone, the gene expression of both cytokines had increased 60–70 fold over the expression of the control, untreated macrophages. While a dose dependent increase in the presence of each cytokine in the media of the cells also occurred, this increase was not significant when compared to the control (Figure 9C,D). As mentioned above, TNFα and IL-6 are the main markers of M1 macrophage phenotype. These markers were found elevated in M1 macrophages as well as M0 macrophages that were treated with 7-*epi*-clusianone (Figure 9A,B). The data showed that the expression of inflammatory genes was augmented when M0 macrophages were exposed to 0.2 µM or higher concentrations. Strikingly, the inflammatory response reaches to the level of M1 macrophages when we exposed M0 macrophages to 20 µM of 7-*epi*-clusianone. These results indicate the potential of 7-*epi*-clusianone to polarize M0 macrophages to anticancer M1 macrophages.

## 3. Discussion

Natural products have been instrumental in the treatment of cancer and are currently garnering renewed interest as lead compounds for cancer therapies and complementary treatments due to the shortcomings of targeted therapeutics. Compounds which simultaneously affect immune regulation of cancer and induce cancer cell death may provide a uniquely well suited complementary treatment to current targeted therapy and chemotherapy regimens. 7-*epi*-clusianone and its configurational isomers, are natural products that have been shown to exhibit a wide array of biological effects. The anticancer effects of 7-*epi*-clusianone have been previously investigated in a limited number of cancer cell lines [34,35,36,37], but the mechanism of action and the molecular targets of the compound have not been fully investigated, nor has its ability to modulate the immune response to cancer.

In this study, 7-*epi*-clusianone was demonstrated to inhibit the growth of cancer cells across 9 tissue types and 60 individual cancer cell lines. A growth inhibitory effect was observed for each cell line, but an LD50 less than the highest concentration tested was only observed in 25 of the 60 cell lines tested. These 25 cell lines were composed of cell lines from each tissue type tested excluding leukemia. In combination, these effects suggest that 7-*epi*-clusianone could be used to slow the progression of many variations of cancer, but it will not be cytotoxic to all types of cells. As 7-*epi*-clusianone is likely to be safe in vivo at relatively high concentrations [30] and the compound is selective in inducing cell death, has the potential to be safely used for the treatment of a diverse group of cancers. In particular, renal cancer, melanoma, central nervous system tumors, colon cancer, and non-small cell lung cancer appear to be sensitive to the cytotoxic effects of 7-*epi*-clusianone. It is unclear what factors determine the sensitivity of each cancer cell line to 7-*epi*-clusianone, and further study will be required to determine how to best predict sensitivity within a particular cancer tissue. 7-*epi*-clusianone’s anticancer effects have the biggest potential for clinical impact due to the high rate of lung cancer related death in the United States. 7-*epi*-clusianone has the ability to induce cell death in non-small cell lung cancer cell lines and inhibit an ALK tyrosine kinase mutation responsible for acquired resistance to treatment.

In order to further investigate the anticancer effect of 7-*epi*-clusianone on non-small cell lung cancer, additional experiments were performed on a lung cancer cell line which had exhibited sensitivity to the compound. The non-small cell lung cancer cell line that had exhibited the greatest sensitivity to 7-*epi*-clusianone was NCIH460. Cell cycle analysis revealed that the cell death was preceded by G1 phase arrest, and a subsequent reduction of S phase and G2/M phase cells. A similar G1 phase arrest has been observed after treatment of A549 lung cancer cells with 7-*epi*-clusianone suggesting a similar mechanisms of action for inducing cytotoxicity across lung cancer cell types [37].

The cell death induced in NCIH460 cells by 7-*epi*-clusianone is hypothesized to proceed through an apoptotic mechanism. As the concentration increased, more annexin V positive cells were observed via flow cytometry. It should be noted that both cells undergoing necrosis and apoptosis may be positive for both PI and Annexin V at late stages of cell death and therefore the Annexin V/PI flow cytometry results in Figure 5 do not conclusively show apoptosis occurred. However, the population of Annexin V positive cells with low PI staining observed with treatment of 7.6 and 15 µM of 7-*epi*-clusianone suggests the presence of cells which are leaving early apoptosis (when the intact cell membrane prevents PI staining) and entering late apoptosis (when the cell membrane begins to permeabilize). This population appears to dissipate at higher concentrations of 7-*epi*-clusianone as the cells enter apoptosis more rapidly.

The apoptosis hypothesis was supported by the cleavage of caspase 9 and PARP, which participate in the caspase cascade leading to cell death after mitochondrial depolarization has occurred [43,44]. Cleavage of caspase 9 and PARP in NCIH460 was evidenced by the decrease in the abundance of full length caspase 9 and PARP and the increase in the abundance of cleaved caspase 9 and PARP as the concentration of 7-*epi*-clusianone was increased. A decrease in full length caspase 7 and caspase 8 expression upon treatment of NCIH460 cells with 7-*epi*-clusianone was also observed. The decrease in full length caspase 7 and 8 was further evidence that the caspase cascade leading to apoptotic cell death had been activated, however the lack of cleaved caspase 7 and 8 observed makes this finding inconclusive. Additionally, whether caspase 9 was activated through the receptor mediated apoptosis pathway or the intrinsic apoptosis pathway was not possible to determine due to the lack of cleaved caspase 7 or 8 [44]. The absence of cleaved caspase 7 or 8 in this study does not, however, rule out the possibility of receptor mediated or intrinsic apoptosis as the cleaved proteins may have been present at earlier incubation time points before being digested by the cell.

In addition to inducing dose dependent apoptosis in non-small cell lung cancer, 7-*epi*-clusianone also inhibited the invasion of NCIH460. Significant invasion inhibition was observed at a concentration as low as 200 nM. Further, 7-*epi*-clusianone was shown to significantly reduce the tube formation between HUVEC endothelial cells at a concentration of 20 µM. Tube formation is a critical step in the process of angiogenesis which is utilized by the wound healing process as well as by cancers to feed tumor growth [45,46,47]. By inhibiting angiogenesis and tumor cell invasion, 7-*epi*-clusianone may act to inhibit continued tumor growth and metastasis of non-small cell lung cancer into healthy tissue. These effects were seen in concentrations less than the concentrations required to induce cell death, suggesting 7-*epi*-clusianone directly inhibits molecular targets associated with angiogenesis and invasion which were outside the scope of this study.

Mechanistic studies were however performed to elucidate the mechanism by which 7-*epi*-clusianone induces cell death in non-small cell lung cancer. For the first time, the direct effect of 7-*epi*-clusianone on cancer proliferation targets including microtubules and ALK (C1156Y) was determined. While 7-*epi*-clusianone has been previously suggested to impact microtubule structure [37], the mechanism of this effect was unclear. This study demonstrated that 7-*epi*-clusianone acts by stabilizing and increasing the rate of polymerization of tubulin directly. This mechanism of action is one shared by chemotherapy agents, such as paclitaxel, though the concentration of 7-*epi*-clusianone required to impact the polymerization of tubulin was high compared to the concentration necessary to induce growth inhibition or cell death on NCIH460 cells. As a result, it is likely that additional molecular targets are influenced by 7-*epi*-clusianone in order to impart its cytotoxic effects. We further determined that 7-*epi*-clusianone directly inhibits the function of two tyrosine kinases, ALK (C1156Y) and JAK3. While these molecular targets are not largely present within NCIH460 lung cancer, and thus are not likely to be involved with a mechanism of cell death, they do suggest 7-*epi*-clusianone may target non-small cell lung cancer cells with acquired resistance to kinase inhibitors.

Due to the increasing role of immune targeted therapies in lung cancer, the ability of 7-*epi*-clusianone to modulate the immune system within the tumor microenvironment was also investigated [48]. Macrophages are immune cells that play a role in T-cell activation in addition to engulfing pathogens and dead cells. Within the tumor microenvironment, tumor associated macrophages (TAMs) can be formed, which resemble macrophages polarized to an M2, anti-inflammatory state and aid in tumor growth and evasion from the immune system [49]. While some studies have suggested eliminating TAMs as a therapeutic strategy, others have hypothesized that polarizing macrophages to the M1 state within the tumor microenvironment will not only eliminate the tumor-supportive functions of TAMs but will also activate the immune system against the tumor [50]. In this study, 7-*epi*-clusianone was shown to increase the gene expression of TNFα and IL-6 within THP-1 derived macrophages, suggesting that cells polarize to an anticancer phase, pro-inflammatory M1 macrophages. This regulation of macrophages suggests that 7-*epi*-clusianone might be a useful complementary treatment when combined with current first-in-line immune therapies. This is especially true in the case of CAR-T cell therapy, in which macrophage dysfunction is the source of some of the most serious side effects [12].

Future studies will be required to determine the effectiveness of 7-*epi*-clusianone as a cancer therapy. More mechanistic studies should be performed to elucidate the primary targets for 7-*epi*-clusianone’s cytotoxic and immune regulatory properties. Of particular interest to future studies should be the effect of 7-*epi*-clusianone on non-small cell lung cancer due the high mortality rates of this malignancy and the compound’s ability to induce apoptosis and inhibit invasion of non-small cell lung cancer cell lines. With this information, the groundwork will be laid to perform in vivo determinations of the efficacy of 7-*epi*-clusianone in inducing cancer cell death and regulating the immune response.

7-*epi*-clusianone is a promising lead compound for anticancer therapies due to its combined apoptotic, anti-angiogenesis, anti-invasion, and immune-regulating properties. The natural compound was shown to have three direct molecular targets in this study, with more targets likely undiscovered. The multi-targeting nature of 7-*epi*-clusianone stands to provide a complementary treatment to emerging targeted oncology therapeutics, which suffer from limited efficacy when used alone as well as toxicities and acquired resistance. Additionally, 7-*epi*-clusianone may combine growth inhibitory and cytotoxic effects on cancer cells with a modulation of the tumor microenvironment to oppose the formation and spread of cancer when used alone. In summary, 7-*epi*-clusianone may provide a robust and multipronged treatment for cancer, especially non-small cell lung cancer.

## 4. Materials and Methods

### 4.1. 7-epi-clusianone Source and Identification

The polycyclic polyprenylated acylphloroglucinol, 7-*epi*-clusianone, was isolated from the aerial parts of *Hypericum scabrum*. Air-dried, powdered aerial parts of *Hypericum scabrum* (5.0 kg) were extracted with *n*-hexane (4 × 20 L) by maceration at room temperature. The extract was concentrated in vacuum to afford dark gummy residue (150 g). The residue was separated on a silica gel column (230−400 mesh, 6 × 75 cm, 800 g) with a gradient of *n*-hexane-EtOAc (100:0 to 0:100) as eluent, followed by increasing concentrations of MeOH (up to 50%) in EtOAc. After screening by TLC, fractions with similar compositions were pooled, to yield 15 combined fractions. From fraction eluted with *n*-hexane–EtOAc (95:5) a yellow crude solid was obtained, which was triturated with MeOH to give 7-*epi*-clusianon as white powder (100 mg). The isolated compound was stored in a sealed vial in the dark, at 4 °C, until used. The structure was elucidated by a combination of 1D and 2D nuclear magnetic resonance (NMR, Bruker, Billerica, MA, USA) and time of flight mass spectrometry, as well as comparing with the data in literature [51]. For NMR analysis, 7-*epi*-clusianone was dissolved in dimethyl sulfoxide (DMSO) and analyzed using a Bruker Avance III-HD 400 MHz. ^1^H-NMR, ^13^C-NMR, H-H correlation spectroscopy (COSY), heteronuclear single quantum coherence (HSQC), and heteronuclear multiple bond correlation (HMBC) spectrums were generated. For mass spectrophotometry analysis, 7-*epi*-clusianone was dissolved in methanol and analyzed using liquid chromatography-mass spectrometry (LC-MS) on a Thermo Orbitrap Velos Pro. NMR and mass spectrometry (MS) data can be found in Figures S1 and S2. For all cell culture experiments, 7-*epi*-clusianone was diluted in DMSO at a concentration of 20 mM before being diluted into the cell culture media specified for each cell line.

### 4.2. Cell Lines and Reagents

The non-small-cell lung cancer cell line, NCIH460, was purchased from the Development Therapeutics Program, Division of Cancer Treatment and Diagnosis tumor repository. NCIH460 cells were maintained in RPMI-1640 media (Corning, Corning, New York) supplemented with 10% Fetal Bovine Essence (FBS; VWR, Radnor, PA, USA) and 2 mM L-glutamine (Sigma Aldrich, St. Louis, MO, USA). THP-1 human monocytic cells were obtained from American Type Culture Collection (ATCC, Manassas, WV, USA). THP-1 cells were maintained in RPMI-1640 media supplemented with 10% FBS and 0.05 mM 2-mercaptoethanol (Sigma Aldrich, St. Louis, MO, USA). Human umbilical vascular endothelial cells (HUVEC) were purchase from Lonza Inc. HUVEC were cultured in endothelial cell basal media 2 (EBM-2, Lonza Inc., Basel, Switzerland) supplemented with endothelial growth medium bulletkit (EGM-2; Lonza, Inc., Basel, Switzerland).

THP-1 cells were differentiated into THP-1 M0 macrophages by culturing the cells with 100 ng/mL of 12-myristate 13-acetate (PMA; Sigma Aldrich, St. Louis, MO, USA) for 24 h. After differentiation, the cells were washed three times with serum free RPMI-1640 medium (Gibco, Dublin, Ireland) to remove non-differentiated cells. To activate, M0 macrophages were treated with 100 ng/mL of lipopolysaccharide (LPS, Sigma Aldrich, St. Louis, MO, USA and 20 ng/mL of interferon gamma (IFN-γ, Peprotech, Rock Hills, NJ, USA) for 24 h. Polarized M0 macrophages were used as a positive control for comparison to groups that were treated with the compound.

### 4.3. NCI-60 Cell Line Screening

Cytotoxicity screening was performed using the National Institute of Health’s (NIH) National Cancer Institute-60 (NCI-60) screening program [52]. This panel utilized a Sulforhodamine B assay to quantify the total cellular protein present after 48 h of treatment with a 7-*epi*-clusianone. The panel screened 60 unique cancer cell lines. After sufficient activity was observed using one dose of 7-*epi*-clusianone (20 µM), the screening was repeated using a five-point dilution. For each of the cell lines, the concentration of compound required to reduce the growth of cells to 50% that of the vehicle control (GI50), the concentration required to inhibit the growth of any amount of cells (TGI), and the concentration required to induce cell death of 50% of the seeded cells (LD50) was determined. A thorough description of the panel and its development is given by Shoemaker [53].

### 4.4. Cytotoxicity Assay (MTS)Assay

In order to determine cell viability, HUVECs and NCIH460 were cultured in their growth media to reach 80% confluency. HUVECs and NCIH460 were seeded at a density of 5 × 10^3^ and 4 × 10^3^ cell per 96 well plates, respectively. For all cell types, 100 μL of cell culture media was used. The cells were then incubated for 24 h at 37 °C and 5% CO_2_ to allow for cell attachment. The culture media was aspirated and media supplemented with the different concentrations of 7-*epi*-clusianone (0.2, 2, and 20 µM) was replaced. The compound was first dissolved at a concentration of 10 mg/mL in DMSO and subsequently diluted in culture media. Following 8 and 24 h incubation for HUVECs and NCIH460, media containing 20% MTS solution was replaced with growth media and incubated for 2 h. The absorbance of each well at 490 nm was measured using a Spectramax 190 spectrometer (Molecular Devices Corporation, Sunnyvale, CA, USA).

### 4.5. In Vitro Invasion Assay

Cell migration of NCIH460 (60 × 10^4^ cells/well) and THP-1 (100,000 cells/well) cells was assessed by making a cell-free gap with a Culture-Insert (IbiTreat, Martinsried, Germany) following manufacturer’s instructions. Twenty-four hours later, the inserts were removed, and cell debris was washed with phosphate buffered saline (PBS). The samples were supplemented with different concentrations of 7-*epi*-clusianone in cell culture media and incubated at 37 °C and 5% CO_2_ for 24 h. Images were taken at different time intervals using a phase contrast Nikon Eclipse Ti-E inverted microscope. Quantification of the percent invasion was performed by measuring the gap distance using the following formula,

(1) $$invasion\;\%=\frac{\;(W_{0}-W_{n})}{W_{0}}*100\%$$

where *W_n_* is the average of three gap width measurements at 6, 12, or 24 h, and *W*_0_ is the initial width of the cell-free gap. The media was removed, and the cells were stained with cell stain solution (Cell Biolabs, Inc. San Diego, CA, USA). Images were taken using a phase contrast inverted microscope (Invitrogen EVOS FL Auto Cell Imaging).

### 4.6. Tube Formation Assay

Growth factor reduced BD Matrigel^TM^ (Corning, Corning, NY, USA) was thawed on ice at 4 °C overnight. Next, 50 µL of Matrigel^TM^ was added to each well of a pre-chilled 96-well plate and incubated at 37 °C and 5% CO2 for 30 min until the Matrigel^TM^ had formed a gel. A suspension of 20,000 HUVEC in 100 µL of cell culture media dosed with 7-*epi*-clusianone was added to each well. The vehicle control consisted of only HUVEC cells and growth media. At the end of the 8-h time point, the cells were stained with cell stain solution as outlined in the in vitro invasion assay protocol. The junctions, or tubes, connecting the endothelial cells were photographed using an Invitrogen EVOS FL Auto at 4× magnification and counted manually.

### 4.7. Cell Cycle Analysis

The effect of 7-*epi*-clusianone on the cell cycle of NCIH460 cells was determined using flow cytometry. The cells were seeded (2500 cells/cm^2^) allowed to attach and treated with 7-*epi*-clusianone. At 6, 12, 24, and 48 h of treatment, the drugged media was collected, and the cells were trypsinized, and centrifuged. The resulting pellet was suspended in 1 mL of ice-cold PBS, which was then added dropwise to 3 mL of ice-cold 70% ethanol in deionized water. The suspension was kept at 4 °C for at least 24 h to allow the cells to fix. Once all time points had been collected, the cells were again centrifuged and the resulting pellets were suspended in FxCycle PI/RNase Staining Solution (Invitrogen, Carlsbad, CA, USA) for 15 min, then analyzed using a BD LSR II flow cytometer. Propidium iodide (PI; Life Technologies, Carlsbad, CA, USA) expression was used to quantify the percentage of cells in the sub-G1, G0/G1, S, and G2/M phases.

### 4.8. Annexin V/PI Apoptosis Assay

The ability of the 7-*epi*-clusianone to induce apoptosis in NCIH460 cells was determined using the FITC Annexin V/ Dead Cell Apoptosis Kit (Invitrogen, Carlsbad, CA, USA). The cells were seeded (2000 cells/cm^2^) allowed to attach and treated with 7-*epi*-clusianone. After 48 h of incubation, the media was collected, and the cells were trypsinized and centrifuged. The supernatant was discarded, and the pellet was washed with ice cold PBS, centrifuged, and resuspended in Annexin V buffer at 1,000,000 cells/mL. Next, 100 µL of the cell suspensions were added to flow cytometry tubes, and 5 µL of Annexin V/FITC antibody and 1 µL of PI working solution were added to each sample. The samples were incubated for 15 min at room temperature and analyzed using a BD LSR II flow cytometer.

### 4.9. Western Blotting

The effect of 7-*epi*-clusianone on apoptosis related proteins in NCIH460 cells was assessed using western blotting. The cells were seeded at a density of 60,000 cell/cm^2^ and allowed to attach for 24 h. Then, the media was replaced with media supplemented with either a vehicle control or the designated concentration of 7-*epi*-clusianone and incubated for 12 and 24 h. The cells were then lysed by suspending the pellet in Radio-immunoprecipitation assay (RIPA; Cell Signaling Technologies, Danvers, Massachusetts) buffer supplemented with phenylmethylsulfonyl fluoride (PMSF; Sigma Aldrich, St, MO, USA) at a concentration of 400 µL/10^7^ cells. The protein was quantified by the bicinchoninic acid (BCA, Cell Signaling Technologies, Danvers, MA, USA) assay following the manufacturer’s protocol.

Lysates (50 μg of protein) were separated by SDS-polyacrylamide gel electrophoresis (SDS-PAGE). Proteins were then transferred to a nitrocellulose membrane (Bio-Rad, Hercules, CA, USA) and incubated with appropriate antibodies.

All primary and secondary antibodies were purchased from Cell Signaling Technologies and were sourced from rabbit. Secondary antibody was anti-rabbit horseradish peroxidase (HRP)-linked. The membrane was washed, then incubated with SignalFire reagent for 2 min and imaged using a BioRad ChemiDoc MP Imaging system. If a membrane was re-probed for a different protein, the membrane was stripped using Restore western blot stripping buffer (Thermo Scientific, Waltham, MA, USA).

### 4.10. Tubulin Polymerization Assay

Tubulin polymerization was assessed using the Tubulin Polymerization Assay Kit (Cytoskeleton, Denver, CO, USA) as per the manufacturer’s instructions. 7-*epi*-clusianone was diluted in DMSO at 20 mM before being diluted to a 10× solution in General Tubulin Buffer. Paclitaxel was used as the positive control. Absorbance correlating to the extent of polymerization was recorded every minute for a total of one hour. Each experimental group was repeated in triplicate.

### 4.11. DiscoverX Kinase Panel

7-*epi*-clusianone was submitted to the DiscoverX KINOMEscan scanTK panel to determine its ability to directly inhibit the function of 135 tyrosine kinases. The assay is an active site-directed competition assay, which does not require the use of ATP to assess kinase function. One concentration of 7-*epi*-clusianone (20 µM) was tested in the panel, and the results were reported as the percent of remaining function for each kinase upon treatment with 7-*epi*-clusianone.

### 4.12. Macrophage Cytotoxicity Assay

Macrophage viability in response to 7-*epi*-clusianone treatment was assessed using CellTitter 96 Aqueous Non-Radioactive Cell Proliferation assay (Promega, Madison, WI, USA). THP-1 cells were seeded into 96-well tissue culture plates (100,000 cells/well) and differentiated to THP-1 M0 macrophages. After cell differentiation, the cells were exposed to various concentrations of 7-*epi*-clusianone for 72 h. Then, the cells were washed with PBS, and culture media supplemented 20% MTS solution (Promega, Madison, WI, USA) was added to the cells. The cells were incubated for 2 h, and the absorbance at 490 nm was measured using a Spectramax 190 microplate reader.

### 4.13. Enzyme-Linked Immunosorbent Assay (ELISA)

THP-1 cells were differentiated to THP-1 M0 macrophages in a 24-well plate (300,000 cells/well) and treated with 7-*epi*-clusianone for 72 h. The concentrations of tumor necrosis factor alpha (TNFα) and interleukin-6 (IL-6) in the THP-1 M0 macrophages media were evaluated using human IL-6 and TNFα tetramethylbenzidine (TMB) enzyme-linked immunosorbent assay (ELISA) development kits (Peprotech, Rocky Hill, New Jersey) according to the manufacturer’s protocol. Colorimetric changes were measured using a SpectraMax 190 microplate spectrophotometer at 450 nm with wavelength correction set at 620 nm. Standard curves for each cytokine were run in parallel to convert the absorbance to concentration in each group.

### 4.14. RNA Extraction and Quantitative Real-Time Polymerase Chain Reaction (rt-PCR)

We assessed the expression of pro-inflammatory genes (IL-6 and TNFα) expressed by THP-1 M0 macrophages in the presence or absence (control) of 7-*epi*-clusianone using rt-PCR. M1 macrophages were used as a positive control to assess M0 macrophage polarization. Total RNA was isolated using the Gene Jet RNA Purification kit (Thermo Scientific, Waltham, MA, USA) according to the manufacturer’s instructions. The RNA was prepared as a template for complementary deoxyribonucleic acid (cDNA) synthesis using the iScript cDNA Synthesis kit (Bio-Rad, Hercules, California). Quantitative rt-PCR analysis was performed with the synthesized cDNA and SYBR Green PCR Supermix (Bio-Rad, Hercules, CA, USA). Gene expression was normalized to the housekeeping gene glyceraldehyde 3-phosphate dehydrogenase (GAPDH) and the control group, non-treated THP-1 M0 macrophages (2^−ΔΔC^). Gene expression values were calculated by using the mean cycle threshold (CT) values of the samples. All primers (Table S3) were synthetized by Integrated DNA Technologies (Integrated DNA Technologies, Coralville, IA, USA).

### 4.15. Statistical Analysis

Statistical analyses were performed with GraphPad Prism 7.03 (GraphPad, La Jolla, CA, USA), using multiple t-test followed by the Holm-Sidak method. Data represent the means of the indicated number of independent experiments. Error bars indicate the standard error of the mean or the standard deviation. *p* < 0.05 was considered to be significant.

## Supplementary Materials

The following are available online, Figure S1: The A) ^1^H-NMR spectrum, B) ^13^C-NMR spectrum, C) H-H COSY spectrum, D) HSQC spectrum, and E) HMBC spectrum of 7-*epi*-clusianone in DMSO-d6, Figure S2: Time of flight mass spectrum determination of 7-*epi*-clusianone, Figure S3: Waterfall plot of the A) GI50, B) TGI, and C) LC50 of 7-*epi*-clusianone for 60 cell lines as determined by the NCI-60 five dose screening assay, Figure S4: The effect 7-*epi*-clusianone on A) the viability of THP-1 macrophages, and B) the percent wound closure, Figure S5: Western blot analysis for the expression of Caspase 3 (A), Caspase 9 (B), Caspase 7 (C), Caspase 8 (D), PARP (E), and GAPDH (F). Table S1: Percent growth of the 60 cell lines examined in the NCI-60 five dose screening method, Table S2: Inhibition of 135 tyrosine kinases treated with 7-*epi*-clusianone determine using the DiscoverX scanTK kinase panel, and Table S3: Primers used for quantitative real-time polymerase chain reaction.
