# Supplementary material for: 7-epi-Clusianone, a Multi-Targeting Natural Product with Potential Chemotherapeutic, Immune-Modulating, and Anti-Angiogenic Properties
Source: Molecules. 2019 Dec 3;24(23):4415. doi: 10.3390/molecules24234415 (PMC6930650; doi:10.3390/molecules24234415)
Supplement: Supplementary file 1 [file molecules-24-04415-s001.pdf]

Supplementary information

7-epi-clusianone, a multi-targeting natural product with potential chemotherapeutic, immune-modulating, and anti-angiogenic properties

Wesley F. Taylor[1], Maria Yanez[1], Sara E. Moghadam[1], Mahdi Moridi Farimani[2], Sara Soroury[3], Samad N. Ebrahimi[2], Marzieh Tabefam[2], and Ehsan Jabbarzadeh[1,4,*]

[1] Department of Chemical Engineering, University of South Carolina, Columbia, SC, 29208, USA. wftaylor@email.sc.edu, yanez@mailbox.sc.edu, and eslambol@mailbox.sc.edu
[2] Department of Phytochemistry, Medicinal Plants and Drugs Research Institute, Shahid Beheshti University, G. C., Evin, Tehran, 19839-69411, Iran. m_moridi@sbu.ac.ir; s_ebrahimi@sbu.ac.ir, and m_tabefam@sbu.ac.ir
[3] Department of Phytochemistry, Faculty of Science, Golestan University, Gorgan, 15759-49138, Iran. sarasoroury@gmail.com
[4] Biomedical Engineering Program, University of South Carolina, Columbia, SC 29208, USA
* Correspondence: ehsan@sc.edu, Tel.: (803) 777-3297

A)

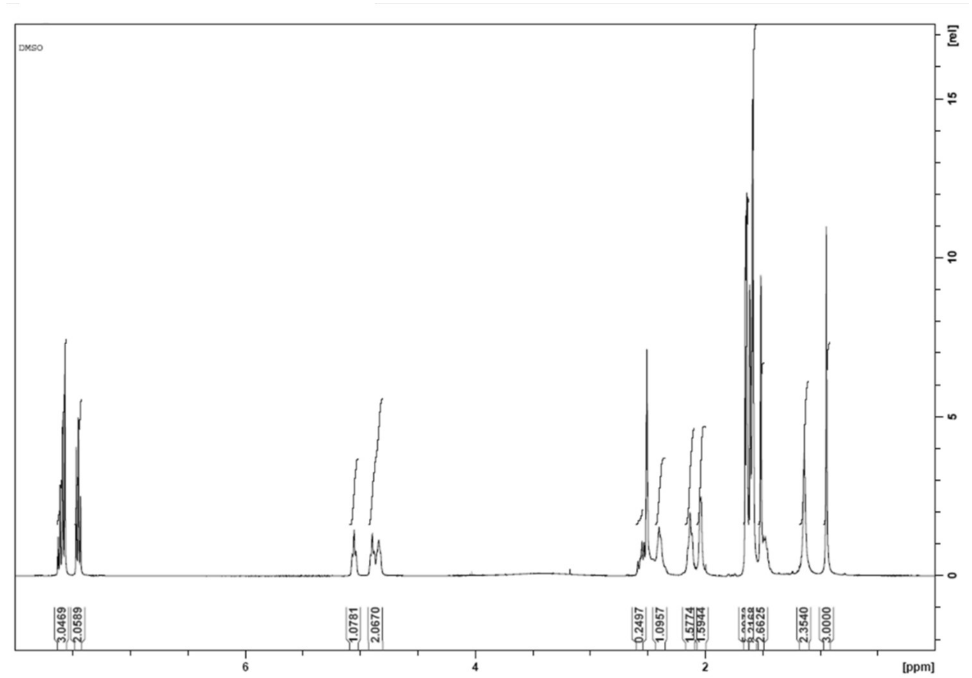

B)

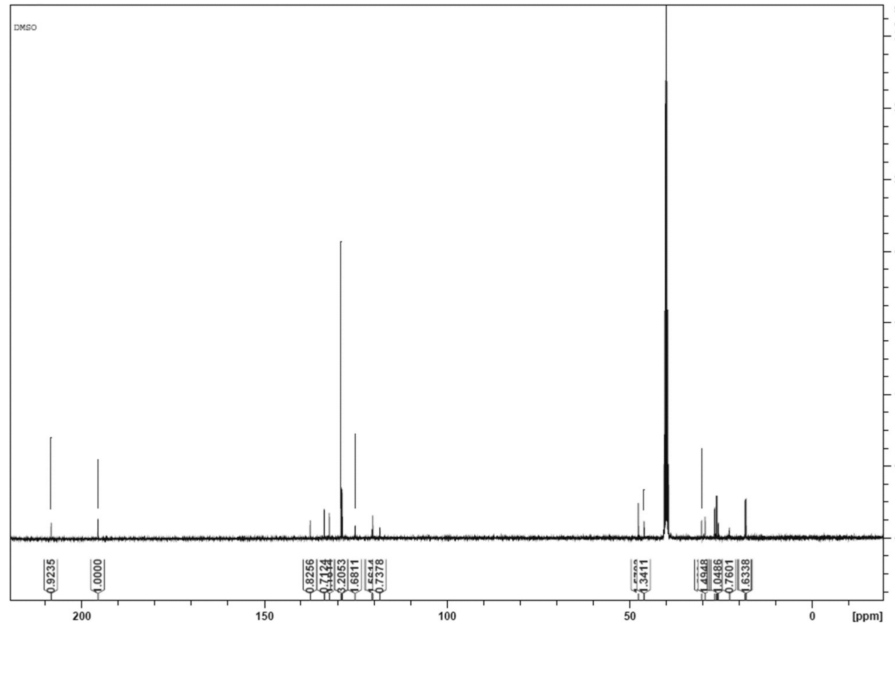

C)

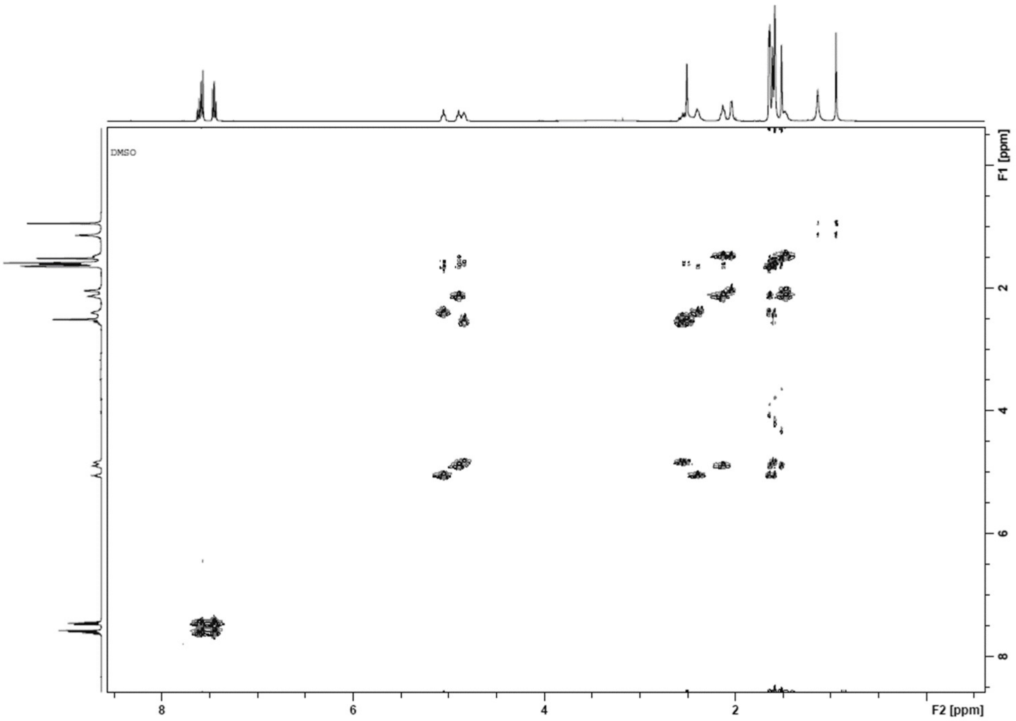
DMSO
F1 [ppm]
2
4
6
8
8
6
4
2
F2 [ppm]

D)

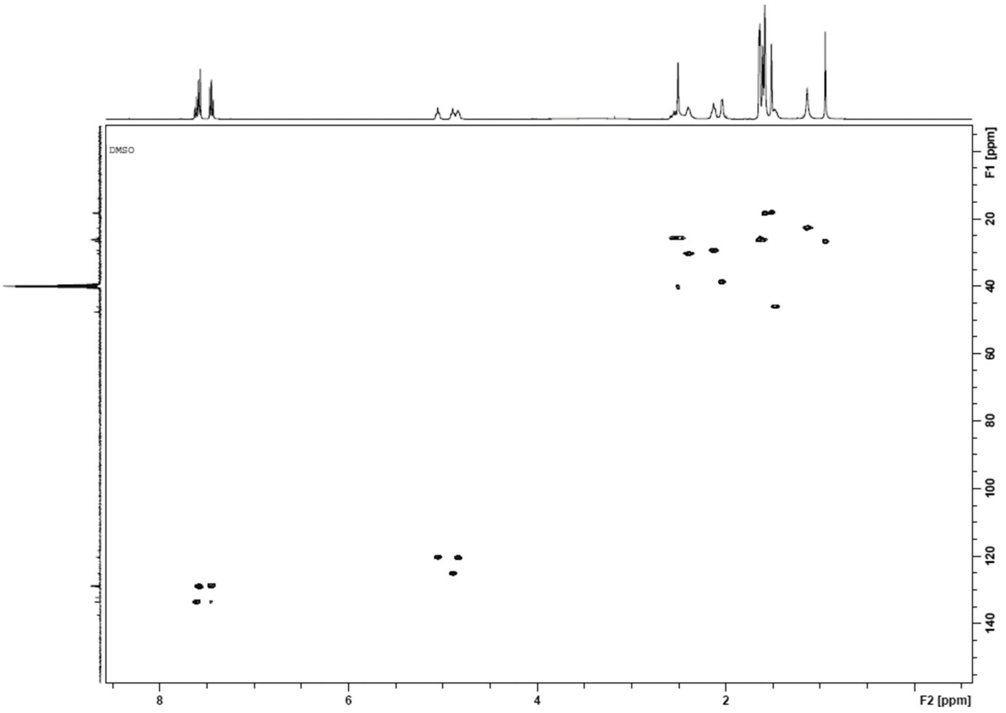
DMSO
F1 [ppm]
20
40
60
80
100
120
140
8
6
4
2
F2 [ppm]

E)

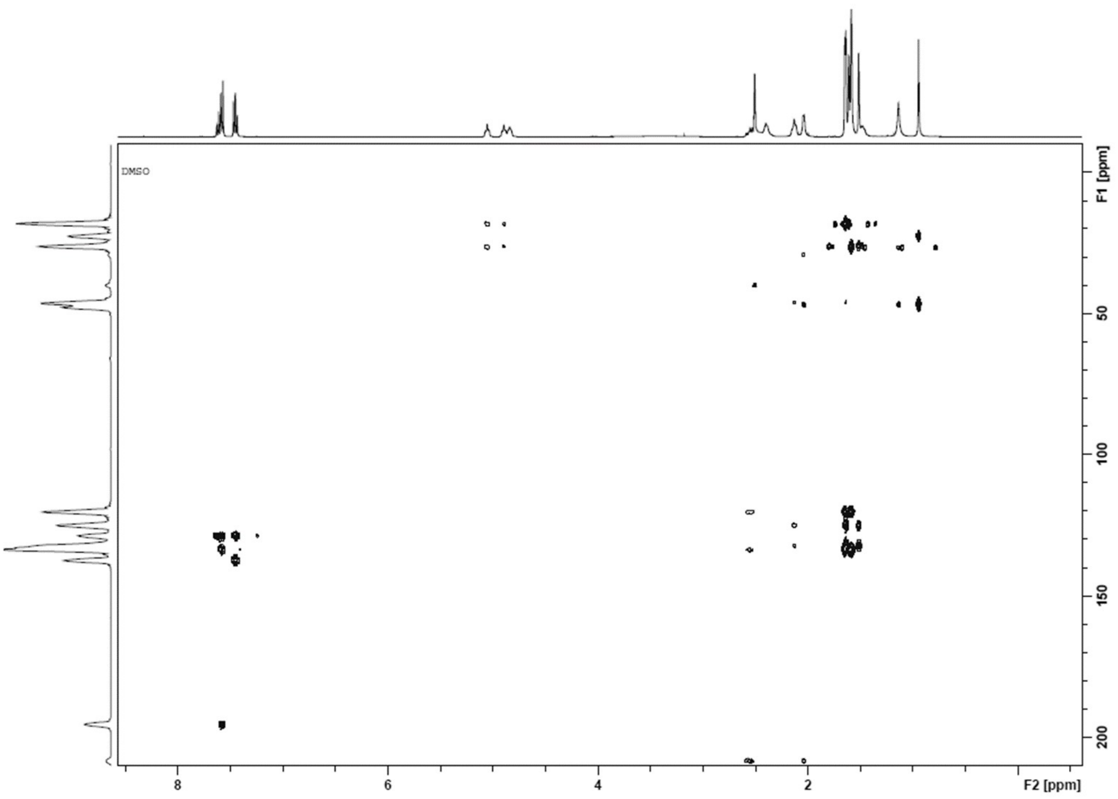

Figure S1. The A) 1H-NMR spectrum, B) 13C-NMR spectrum, C) H-H COSY spectrum, D) HSQC spectrum, and E) HMBC spectrum of 7-*epi*-clusianone in DMSO.

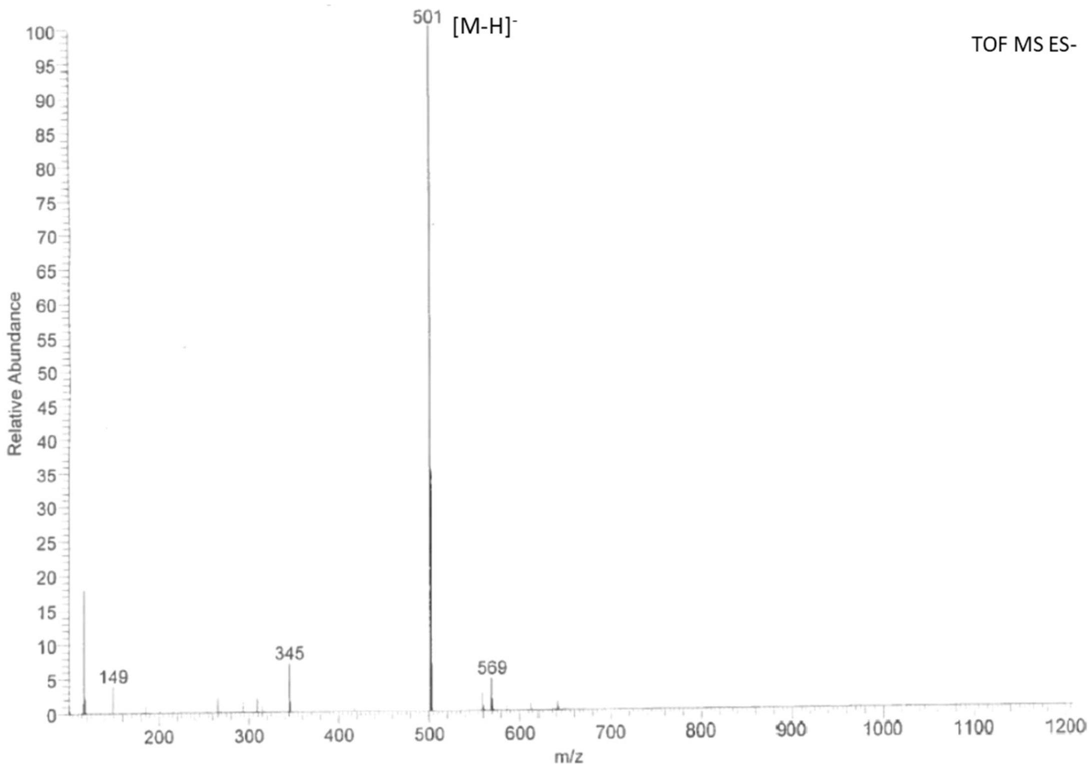

Figure S2: Time of flight mass spectrum determination of molecular weight for 7-*epi*-clusianone.

A)

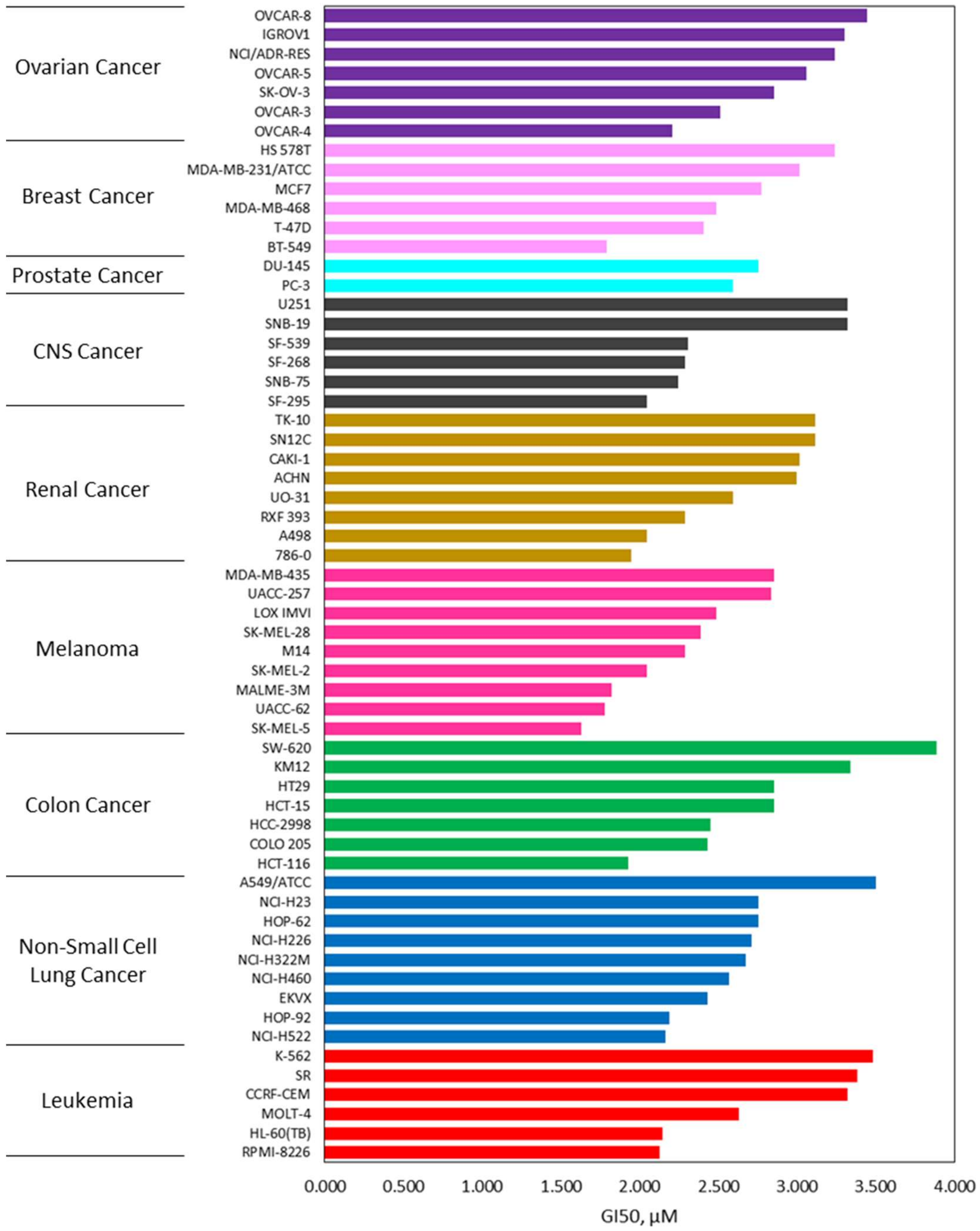

Ovarian Cancer
Breast Cancer
Prostate Cancer
CNS Cancer
Renal Cancer
Melanoma
Colon Cancer
Non-Small Cell Lung Cancer
Leukemia
OVCAR-8
IGROV1
NCI/ADR-RES
OVCAR-5
SK-OV-3
OVCAR-3
OVCAR-4
HS 578T
MDA-MB-231/ATCC
MCF7
MDA-MB-468
T-47D
BT-549
DU-145
PC-3
U251
SNB-19
SF-539
SF-268
SNB-75
SF-295
TK-10
SN12C
CAKI-1
ACHN
UO-31
RXF 393
A498
786-0
MDA-MB-435
UACC-257
LOX IMVI
SK-MEL-28
M14
SK-MEL-2
MALME-3M
UACC-62
SK-MEL-5
SW-620
KM12
HT29
HCT-15
HCC-2998
COLO 205
HCT-116
A549/ATCC
NCI-H23
HOP-62
NCI-H226
NCI-H322M
NCI-H460
EKVX
HOP-92
NCI-H522
K-562
SR
CCRF-CEM
MOLT-4
HL-60(TB)
RPMI-8226
0.000
0.500
1.000
1.500
2.000
2.500
3.000
3.500
4.000
GI50, μM

B)

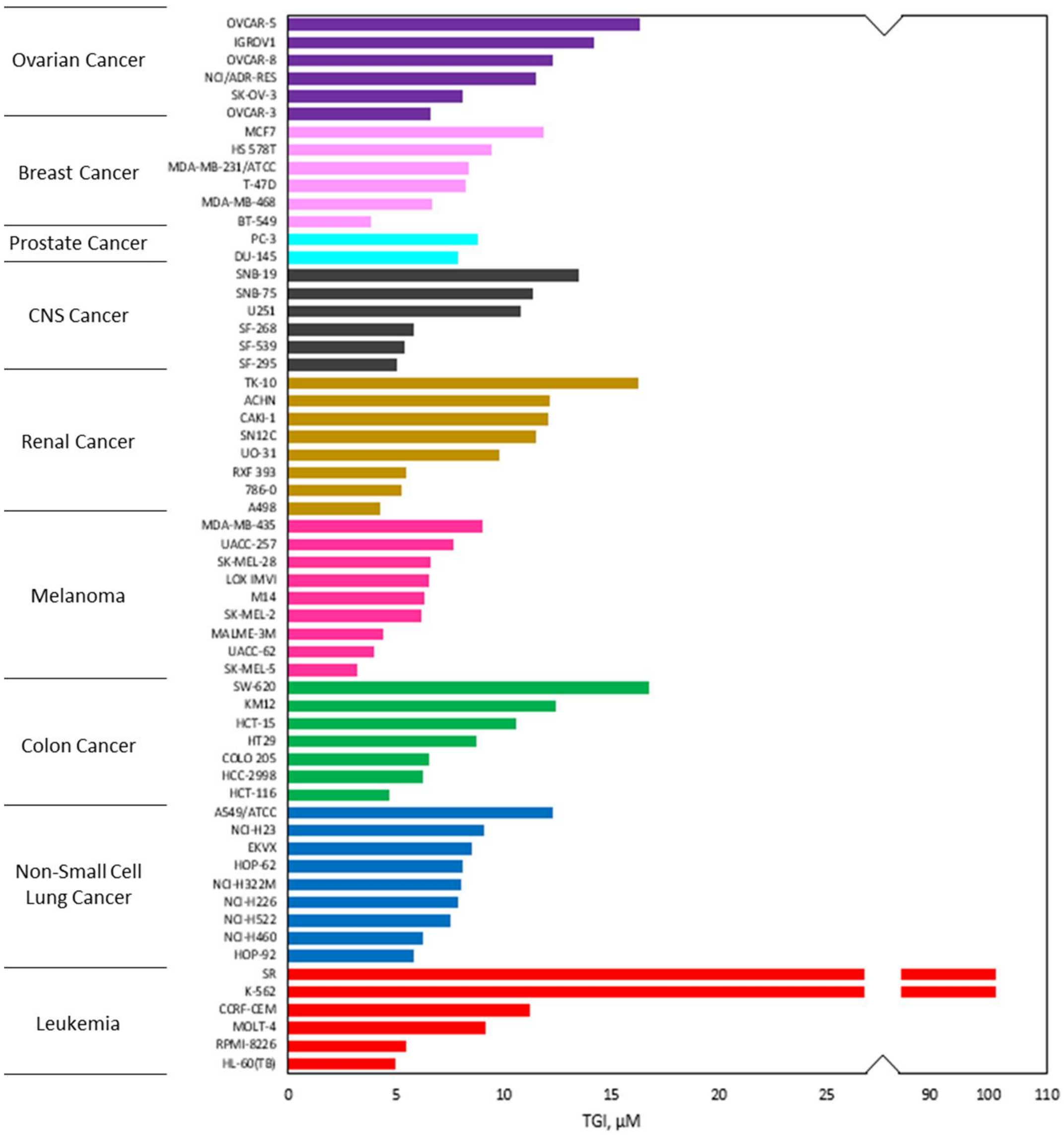

Ovarian Cancer
Breast Cancer
Prostate Cancer
CNS Cancer
Renal Cancer
Melanoma
Colon Cancer
Non-Small Cell Lung Cancer
Leukemia
OVCAR-5
IGROV1
OVCAR-8
NCI/ADR-RES
SK-OV-3
OVCAR-3
MCF7
HS 578T
MDA-MB-231/ATCC
T-47D
MDA-MB-468
BT-549
PC-3
DU-145
SNB-19
SNB-75
U251
SF-268
SF-539
SF-295
TK-10
ACHN
CAKI-1
SN12C
UO-31
RXF 393
786-0
A498
MDA-MB-435
UACC-257
SK-MEL-28
LOX IMVI
M14
SK-MEL-2
MALME-3M
UACC-62
SK-MEL-5
SW-620
KM12
HCT-15
HT29
COLO 205
HCC-2998
HCT-116
A549/ATCC
NCI-H23
EKVX
HOP-62
NCI-H322M
NCI-H226
NCI-H522
NCI-H460
HOP-92
SR
K-562
CCRF-CEM
MOLT-4
RPMI-8226
HL-60(TB)
0
5
10
15
20
25
90
100
110
TGI, µM

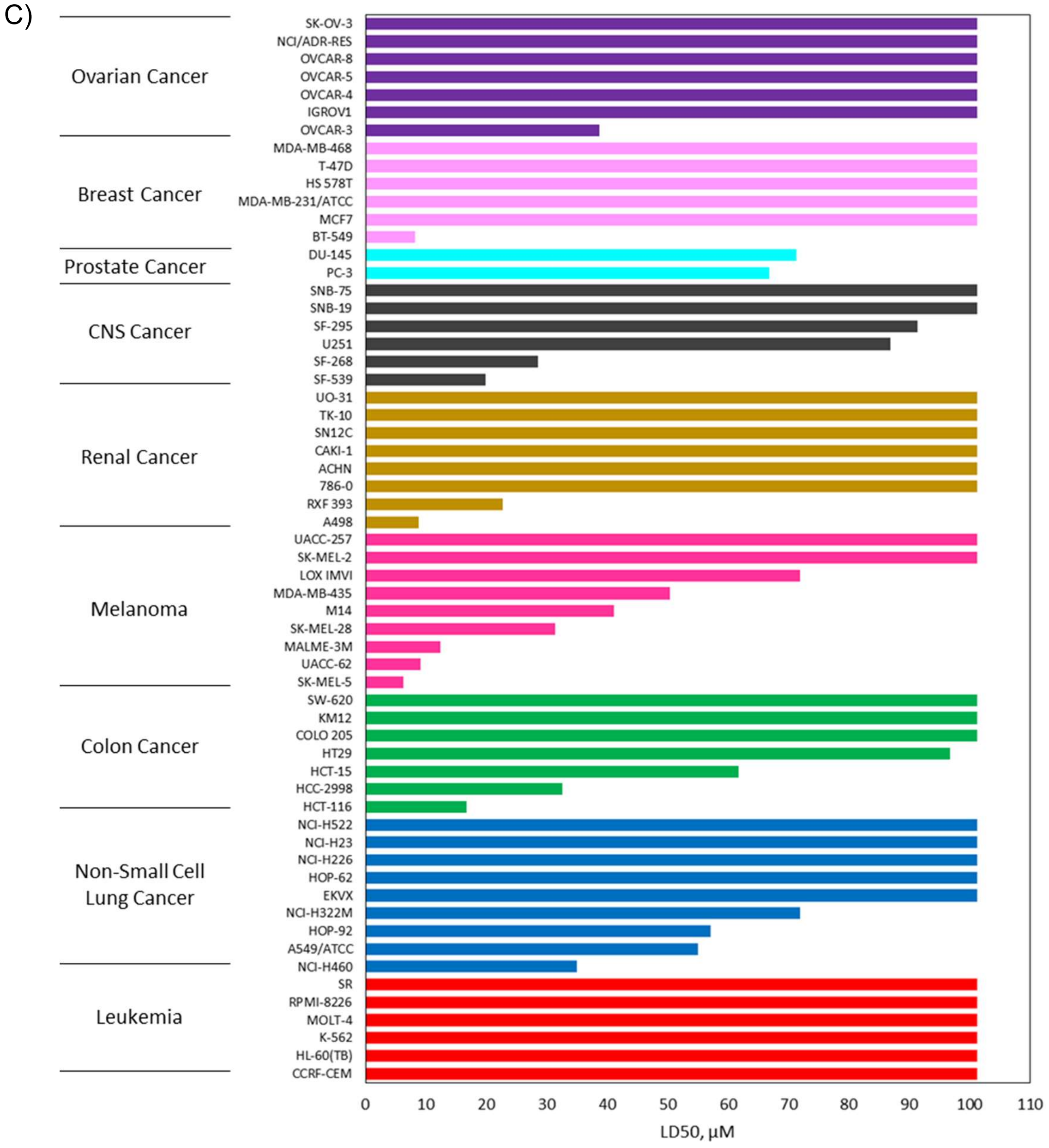

Figure S3. Waterfall plot of the A) GI50, B) TGI, and C) LC50 of 7-*epi*-clusianone for 60 cell lines as determined by the NCI-60 five dose screening assay. The tissue type of each cell line is denoted by color (Purple – Ovarian Cancer, Pale pink – Breast Cancer, Aqua – Prostate Cancer, Grey – CNS cancer, Gold – Renal Cancer, Bright Pink – Melanoma, Green – Colon Cancer, Blue – Non-small cell lung cancer, Red – Leukemia). The GI50, TGI, and LC50 was set to 100 µM if the true concentration was higher than what was tested in the five dose screen.

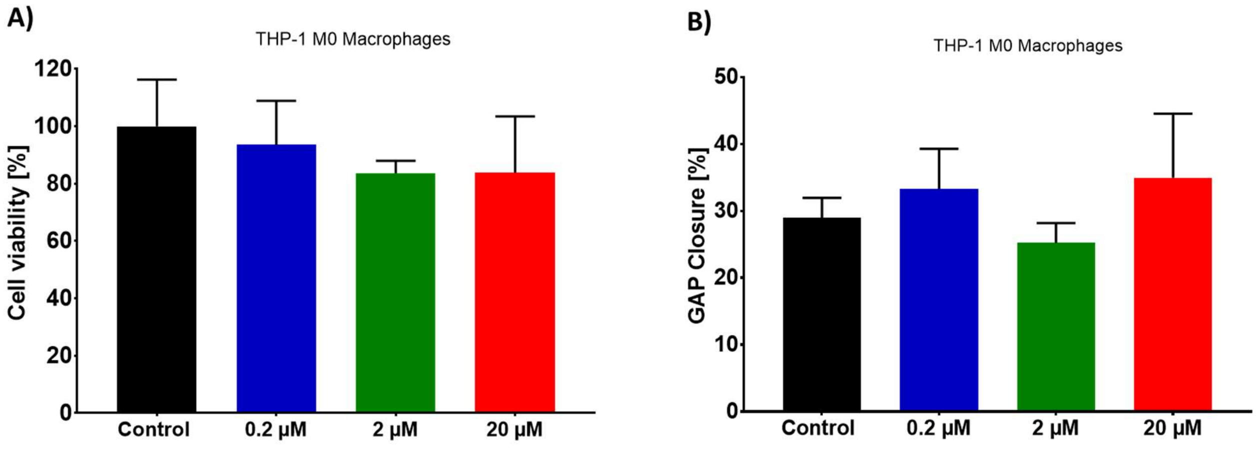

Figure S4. The effect 7-*epi*-clusianone on A) the viability of THP-1 macrophages, as determined by the MTS assay, after 72 hours of exposure to a vehicle control or 0.2, 2, or 20 µM of 7-*epi*-clusianone, and B) the percent wound closure of THP-1 macrophages into a cell-free gap after 24-hour exposure to a vehicle control or 0.2, 2, or 20 µM of 7-*epi*- clusianone.

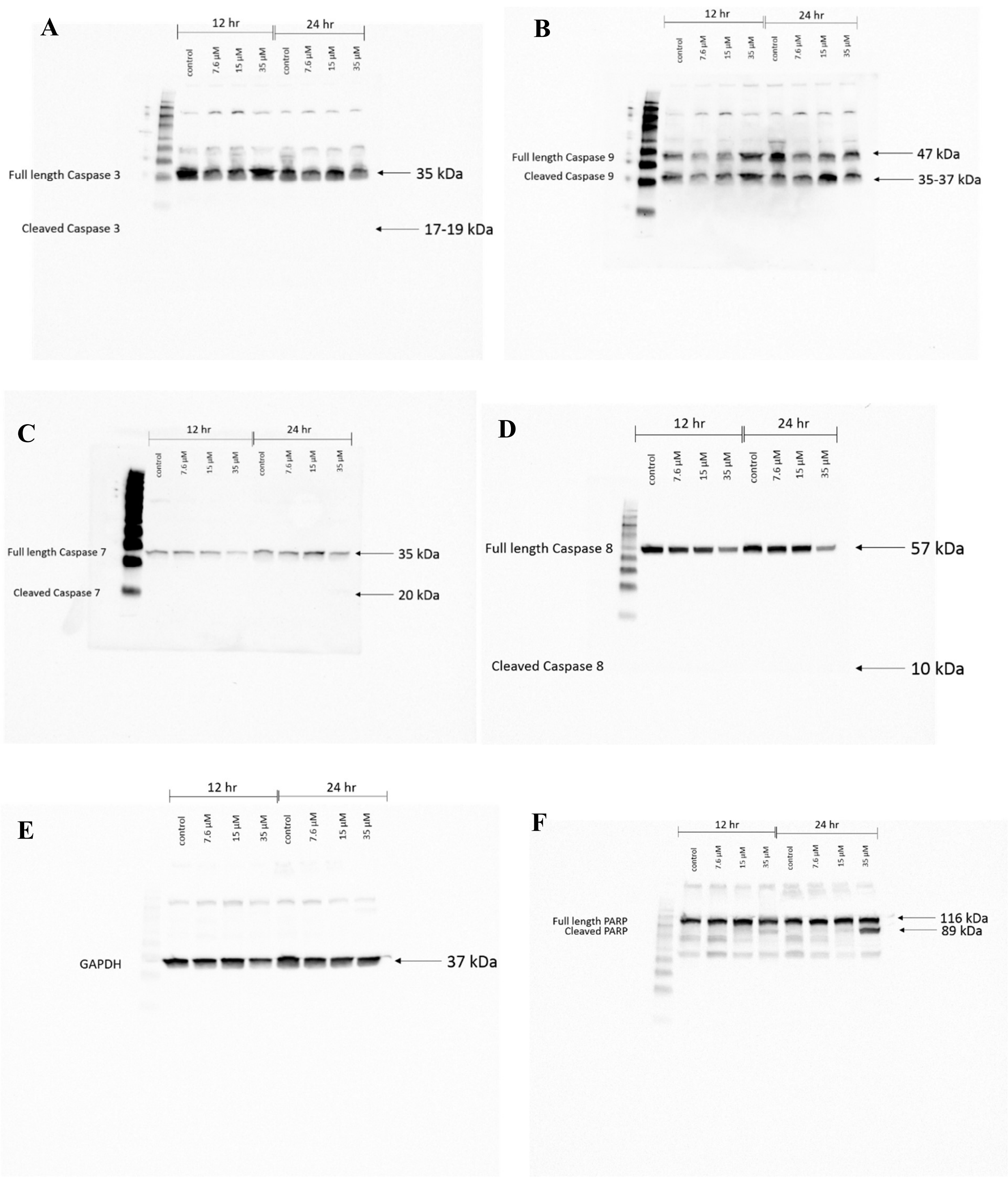

Figure S5. Samples were processed and western blotted for the expression of Caspase 3 (A), Caspase 9 (B), Caspase 7 (C), Caspase 8 (D), PARP (E), and GAPDH (F). Images were taken from the same sample run in duplicates and 2 different gels. The contrast and brightness of the images were adjusted using Image Lab software from Bio-Rad.

Table S1. Percent growth of the 60 cell lines examined in the NCI-60 five dose screening method.

The cancer cell lines are organized by tissue type.

**Leukemia (% Growth)**					
**CCRF-CEM**	103	102	103		-7
**HL-60 (TB)**	94	94	95	-43	-38
**K-562**	98	91	96	10	15
**MOLT-4**	95	96	89	-4	-1
**RPMI-8226**	96	101	90	-33	-34
**SR**	94	99	100	5	1
**Concentration (logM)**	-8.0	-7.0	-6.0	-5.0	-4.0

**Non-Small Cell Lung Cancer (% Growth)**					
**A549/ATCC**	96	103	101	6	-70
**EKVX**	87	90	85	-7	-34
**HOP-62**	92	95	96	-10	-44
**HOP-92**	98	102	89	-29	-57
**NCI-H226**	98	104	96	-12	-44
**NCI-H23**	96	95	92	-5	-36
**NCI-H322M**	96	94	94	-10	-57
**NCI-H460**	107	110	103	-28	-69
**NCI-H522**	93	86	81	-12	-21
**Concentration (logM)**	-8.0	-7.0	-6.0	-5.0	-4.0

**Colon Cancer (% Growth)**					
**COLO 205**	97	98	94	-22	-42
**HCC-2998**	94	103	97	-26	-73
**HCT-116**	92	94	87	-44	-71
**HCT-15**	94	97	90	1	-64
**HT29**	98	97	97	-7	-51
**KM12**	100	101	99	4	-40
**SW-620**	106	106	104	12	-41
**Concentration (logM)**	-8.0	-7.0	-6.0	-5.0	-4.0

CNS Cancer (% Growth)					
**SF-268**	97	95	94	-29	-75
**SF-295**	85	91	89	-39	-51
**SF-539**	88	93	99	-38	-80
**SNB-19**	98	104	100	3	-20
**SNB-75**	72	79	75	3	-48
**U251**	106	104	102	2	-54
**Concentration (logM)**	-8.0	-7.0	-6.0	-5.0	-4.0

Melanoma (% Growth)					
**LOX IMVI**	95	96	96	-22	-55
**MALME-3M**	89	95	83	-47	-79
**M14**	90	95	90	-23	-67
**MDA-MB-435**	92	96	95	-5	-70
**SK-MEL-2**	93	92	82	-23	-38
**SK-MEL-28**	90	95	92	-21	-80
**SK-MEL-5**	94	96	85	-86	-92
**UACC-257**	100	108	102	-14	-32
**UACC-62**	96	96	85	-57	-73
**Concentration (logM)**	-8.0	-7.0	-6.0	-5.0	-4.0

Ovarian Cancer (% Growth)					
**IGROV1**	102	98	97	6	-33
**OVCAR-3**	103	100	97	-22	-70
**OVCAR-4**	89	99	80	-9	5
**OVCAR-5**	88	91	87	10	-37
**OVCAR-8**	107	106	106	1	-8
**NCI/ADR-RES**	100	99	99	2	-31
**SK-OV-3**	95	100	99	-11	-8
**Concentration (logM)**	-8.0	-7.0	-6.0	-5.0	-4.0

Renal Cancer (% Growth)					
**786-0**	86	93	83	-33	-38
**A498**	100	103	98	-60	-51
**ACHN**	93	99	93	1	-13
**CAKI-1**	88	93	92	3	-37
**RXF 393**	101	101	97	-35	-77
**SN12C**	101	99	96	2	-31
**TK-10**	92	88	93	5	-21
**UO-31**	88	88	85	-1	-18
**Concentration (logM)**	-8.0	-7.0	-6.0	-5.0	-4.0

Prostate Cancer (% Growth)					
**PC-3**	93	94	88	-6	-60
**DU-145**	100	100	97	-12	-57
**Concentration (logM)**	-8.0	-7.0	-6.0	-5.0	-4.0

Breast Cancer (% Growth)					
**MCF7**	86	88	88	1	-11
**MDA-MB-231/ATCC**	102	110	103	-9	-42
**HS 578T**	101	106	105	-3	-18
**BT-549**	94	100	88	-64	-80
**T-47D**	79	89	85	-8	-14
**MDA-MB-468**	99	100	96	-21	-44
**Concentration (logM)**	-8.0	-7.0	-6.0	-5.0	-4.0

Table S2. Inhibition of 135 tyrosine kinases treated with 20 μM of 7-*epi*-clusianone determine using the DiscoverX scanTK kinase panel. The panel uses an active site-directed competition assay, which does not require the use of ATP to assess kinase function. Data is presented as a percentage of function after treatment as compared to a vehicle control. A kinase with a remaining function of 30% or less is considered to be significantly inhibited. The significantly inhibited kinases, JAK3 and ALK (C1156Y), are highlighted.

Target	Clusianone	Target	Clusianone
**Gene Symbol**	**%Ctrl 20μM**	**Gene Symbol**	**%Ctrl 20μM**
ABL1(E255K)-phosphorylated	86	CSF1R-autoinhibited	64
ABL1(F317I)-nonphosphorylated	58	CSK	96
ABL1(F317I)-phosphorylated	66	CTK	87
ABL1(F317L)-nonphosphorylated	98	DDR1	86
ABL1(F317L)-phosphorylated	73	DDR2	61
ABL1(H396P)-nonphosphorylated	71	EGFR	88
ABL1(H396P)-phosphorylated	80	EGFR(E746-A750del)	88
ABL1(M351T)-phosphorylated	65	EGFR(G719C)	97
ABL1(Q252H)-nonphosphorylated	84	EGFR(G719S)	98
ABL1(Q252H)-phosphorylated	87	EGFR(L747-E749del, A750P)	90
ABL1(T315I)-nonphosphorylated	53	EGFR(L747-S752del, P753S)	70
ABL1(T315I)-phosphorylated	69	EGFR(L747-T751del,Sins)	96
ABL1(Y253F)-phosphorylated	79	EGFR(L858R)	95
ABL1-nonphosphorylated	80	EGFR(L858R,T790M)	71
ABL1-phosphorylated	88	EGFR(L861Q)	97
ABL2	100	EGFR(S752-I759del)	93
ALK	68	EGFR(T790M)	72
ALK(C1156Y)	30	EPHA1	100
ALK(L1196M)	41	EPHA2	82
AXL	80	EPHA3	94
BLK	95	EPHA7	99
BMX	98	EPHA8	100
BRK	92	EPHB1	97
BTK	70	EPHB2	89

Target	Clusianone	Target	Clusianone
Gene Symbol	%Ctrl 20µM	Gene Symbol	%Ctrl 20µM
EPHB4	92	KIT(D816V)	80
EPHB6	69	KIT(L576P)	73
ERBB2	59	KIT(V559D)	80
ERBB3	93	KIT(V559D,T670I)	85
ERBB4	100	KIT(V559D,V654A)	97
FAK	85	KIT-autoinhibited	78
FER 5	97	LCK	100
FES 9	92	LTK	70
FGFR1	100	LYN	84
FGFR2	90	MERTK	87
FGFR3	94	MET	86
FGFR3(G697C)	100	MET(M1250T)	92
FGFR4	85	MET(Y1235D)	76
FGR 7	85	MST1R	94
FLT1	90	MUSK	95
FLT3	100	PDGFRA	46
FLT3(D835H)	100	PDGFRB	69
FLT3(D835V)	89	PYK2	91
FLT3(D835Y)	87	RET	83
FLT3(ITD)	88	RET(M918T)	100
FLT3(ITD,D835V)	87	RET(V804L)	100
FLT3(ITD,F691L)	82	RET(V804M)	97
FLT3(K663Q)	90	ROS1	68
FLT3(N841I)	100	SRC	90
FLT3(R834Q)	60	SRMS	73
FLT3-autoinhibited	45	SYK	97
FLT4	99	TEC	100
FRK	96	TIE1	100
FYN	98	TIE2	95
HCK	97	TNK1	97
IGF1R	83	TNK2	96
INSR	71	TRKA	93
INSRR	99	TRKB	54
ITK	100	TRKC	100
JAK1(JH1domain-catalytic)	75	TXK	95
JAK1(JH2domain-pseudokinase)	87	TYK2(JH1domain-catalytic)	83
JAK2(JH1domain-catalytic)	76	TYK2(JH2domain-pseudokinase)	92
JAK3(JH1domain-catalytic)	25	TYRO3	96
KIT	76	VEGFR2	58
KIT(A829P)	86	YES	85
KIT(D816H)	96	ZAP70	78

Table S3. Primers used for quantitative real-time polymerase chain reaction.

Gene	5’-3’ primer sequences: (F: forward R: reverse)
TNFα	F: CTG CTG CAC TTT GGA GTG AT
	R: AGA TGA TCT GAC TGC CTG GG
IL-6	F: AGC CAC TCA CCT CTT CAG AAC
	R: GCC TCT TTG CTG CTT TCA CAC
GAPDH	F: GTG GAC CTG ACC TGC CGT CT
	R: GGA GGA GTG GGT GTC GCT GT
